# Clinical Forms of Chikungunya in Gabon, 2010

**DOI:** 10.1371/journal.pntd.0001517

**Published:** 2012-02-14

**Authors:** Dieudonné Nkoghe, Roland Fabrice Kassa, Mélanie Caron, Gilda Grard, Illich Mombo, Branly Bikié, Christophe Paupy, Pierre Becquart, Ulrich Bisvigou, Eric Maurice Leroy

**Affiliations:** 1 Unité des Maladies Virales Emergentes, Centre International de Recherches Médicales de Franceville-CIRMF, Franceville, Gabon; 2 Ministère de la Santé Publique, Libreville, Gabon; 3 Centre National de la Recherche Scientifique et Technologique, Libreville, Gabon; 4 MIVEGEC (IRD 224/CNRS 5290/UM1/UM2), Montpellier, France; 5 Faculté de Médecine, Université des Sciences de la Santé, Libreville, Gabon; Tulane School of Public Health and Tropical Medicine, United States of America

## Abstract

**Background:**

Chikungunya virus (CHIKV) has caused multiple outbreaks in tropical and temperate areas worldwide, but the clinical and biological features of this disease are poorly described, particularly in Africa. We report a prospective study of clinical and biological features during an outbreak that occurred in Franceville, Gabon in 2010.

**Methodology/Principal Findings:**

We collected, in suspect cases (individuals presenting with at least one of the following symptoms or signs: fever, arthralgias, myalgias, headaches, rash, fatigue, nausea, vomiting, diarrhea, bleeding, or jaundice), blood samples, demographic and clinical characteristics and outcome. Hematological and biochemical tests, blood smears for malaria parasites and quantitative PCR for CHIKV then dengue virus were performed. CHIKV+ patients with concomitant malaria and/or dengue were excluded from the study. From May to July 2010, data on 270 laboratory-confirmed CHIK patients were recorded. Fever and arthralgias were reported by respectively 85% and 90% of patients, while myalgias, rash and hemorrhage were noted in 73%, 42% and 2% of patients. The patients were grouped into 4 clinical categories depending on the existence of fever and/or joint pain. On this basis, mixed forms accounted for 78.5% of cases, arthralgic forms 12.6%, febrile forms 6.7% and unusual forms (without fever and arthralgias) 2.2%. No cases of organ failure or death were reported. Elevated liver enzyme and creatinine levels, anemia and lymphocytopenia were the predominant biological abnormalities, and lymphocytopenia was more severe in patients with high viral loads (p = 0.01).

**Conclusions/Significance:**

During CHIK epidemics, some patients may not have classical symptoms. The existence of unusual forms and the absence of severe forms of CHIK call for surveillance to detect any change in pathogenicity.

## Author Summary

Chikungunya fever (CHIK) is a disease caused by a virus transmitted to humans by infected mosquitos. The virus is responsible for multiple outbreaks in tropical and temperate areas worldwide, and is now a global concern. Clinical and biological features of the disease are poorly described, especially in Africa, where the disease is neglected because it is considered benign. During a recent CHIK outbreak that occurred in southeast Gabon, we prospectively studied clinical and biological features of 270 virologically confirmed cases. Fever and arthralgias were the predominant symptoms. Furthermore, variable and distinct clinical pictures including pure febrile, pure arthralgic and unusual forms (neither fever nor arthralgias) were detected. No severe forms or deaths were reported. These findings suggest that, during CHIK epidemics, some patients may not have classical symptoms (fever and arthralgias). Local surveillance is needed to detect any changes in the pathogenicity of this virus.

## Introduction

Chikungunya fever (CHIK) is a neglected tropical disease caused by the Chikungunya virus (CHIKV), an arthropod-borne virus belonging to the genus *Alphavirus* of the *Togaviridae* family. This virus is transmitted to humans via the bite of infected *Aedes* mosquitoes (*Aedes aegypti, Aedes albopictus*). The genome consists of a single positive strand of RNA that encodes four nonstructural proteins involved in virus replication and pathogenesis, and five structural proteins that compose the virion [1]. CHIKV is subdivided into three genotypes based on phylogenetic analyses. These genotypes, based on the gene sequences of an envelope protein (E1), are Asian, East/Central/South African (ECSA), and West African [2]–[6].

Over the past two decades, this virus has caused multiple outbreaks worldwide, particularly in tropical and subtropical areas. Since its initial isolation in Tanzania in 1953, sporadic cases and numerous outbreaks have been reported in Africa, India Ocean Islands, India, and even in Italy, a temperate region. CHIKV circulated in West and East Africa at low levels until 1999–2000, when an outbreak occurred in the Democratic Republic of the Congo (DRC) with around 50,000 cases [7]. From 2004, successive epidemics occurred, starting in Kenya with 13,500 cases and then spreading through the Indian Ocean region, including the Comoros Islands, Mauritius, Mayotte, La Réunion Island, Madagascar and the Seychelles [8]–[13]. On La Réunion Island alone, which has a population of 760,000 inhabitants, at least 266,000 cases were reported [14]. Thereafter, the epidemic arrived on the Indian subcontinent in 2006–2007 and caused more than 1.3 million cases [15]. Genetic analyses showed that the ECSA genotype was responsible for these outbreaks [8], [16]. Numerous cases were subsequently reported all over the world [17], directly associated with the return of tourists from India and affected India Ocean islands [2]. The first reported European outbreak occurred in two contiguous villages of northeastern Italy [18].

A unnoticed and retrospectively diagnosed large outbreak hit Cameroon in 2006 [19], then Gabon one year later, where a concomitant CHIKV/dengue virus- serotype 2 (DENV-2) epidemic raged in the northwest and north; 20,000 cases were recorded and *Aedes albopictus* was identified as the main vector [20]. Isolates from the 2007 Gabon outbreak belonged to the ECSA phylogroup and harbor the A226V mutation [6].

Thus, the virus has proven able to expand to novel ecological niches, together with the vector *Aedes albopictus* [21], [22]. Recently, autochthonous cases of dengue fever (DF) and CHIK were reported in southern France, where *Aedes albopictus* has also been detected, raising serious concerns [23].

CHIK, a word from the Bantu language, means “that which contorts or bends up” referring to the stooped posture that develops in infected patients due to severe joint pain and impaired walking ability.

The clinical manifestations of CHIK are now well described. The infection is characterized by three distinct forms: asymptomatic, classical and severe. The asymptomatic form is revealed by serology, in naïve populations. In its classical form, the illness appears as a “dengue-like” disease, sometimes being confused with DF, particularly in areas where the two viruses cocirculate [3], [22], [24]. After an incubation period of 3–7 days, symptoms start abruptly with acute fever, followed by severe and often debilitating polyarthralgias sometimes lasting months or years. Additional symptoms include a maculopapular rash, myalgias and headaches [3], [22], [24]. In La Reunion Island, severe forms were reported, mainly in patients with underlying medical conditions. These forms included neurological and cardiovascular disorders, acute hepatitis, skin diseases, and respiratory and renal failure. Miscarriages and neonatal infections were also reported, and some deaths were directly attributed to CHIKV [3], [14], [25], [26].

In previous studies, clinical and biological descriptions were mostly retrospective, and included a limited number of patients. Furthermore, except for the first known CHIKV outbreak in Tanganyika [27], most studies were conducted in Asia, the Indian Ocean Islands and Europe [18], [25], [28], [29], while none have concerned Africa, where multiple pathogens co-circulate [7], [19], [20], [30]–[32].

Here, we report the findings of a prospective, exhaustive clinical and biological study of 270 laboratory-confirmed cases during the simultaneous CHIKV/DENV outbreak which occurred in Gabon, a Central African country, in 2010 [33], focusing on the high clinical variability.

## Materials and Methods

### Outbreak area

**Figure 1:**
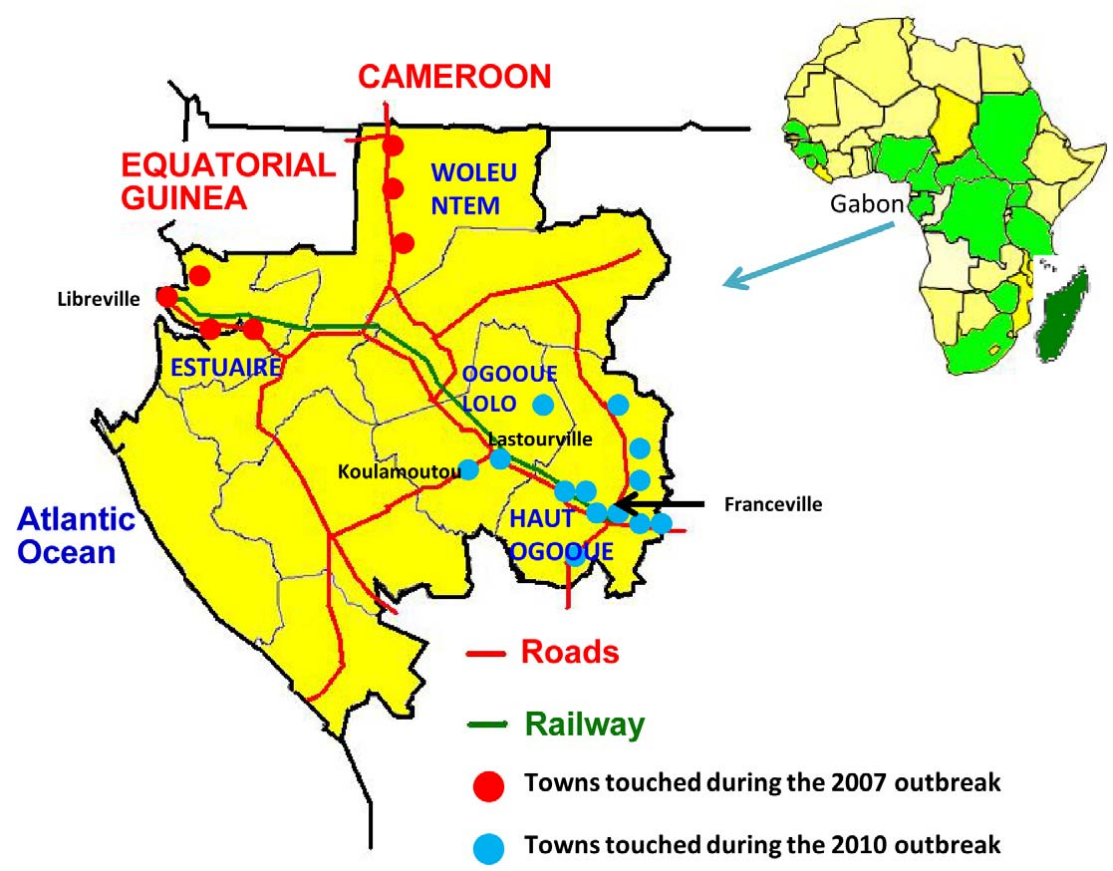
Map of Gabon, and location of the different Chikungunya outbreaks from 2007 to 2010.

A simultaneous CHIKV/DENV outbreak was reported in two provinces (Ogooue Lolo and Haut Ogooue) of southeast Gabon, from April to July 2010. This study took place in Franceville, the main town of Haut Ogooue province, located 512 km south-east from Libreville (capital of Gabon) (Figure 1). In Franceville, there are 4 healthcare capacities, including 2 public hospitals with a total of 170 beds. The Centre International de Recherches Medicales de Franceville (CIRMF), which includes a medical unit, is located in the heart of the city.

### Ethical considerations

The CIRMF team partnered the Ministry of Health (MoH) response team during this outbreak. The investigations (epidemiological and clinical inquiries, blood sampling for laboratory confirmation) were thus considered as part of the public health response. According to the MoH's directives, written consent was not required due to emergency diagnosis. An oral consent was obtained for each patient during interviews. The study was approved by the Regional Health Director, including individual oral consent for blood sampling (Authorization n°189, Figure S1). The results were transmitted to patients and MoH.

### Study population and clinical examination

The study was conducted in the field by two doctors who investigated cases in all healthcare facilities of Franceville, while free medical consultations were provided by another in the CIRMF medical unit.

At the time of this outbreak, all the hospitals were requested to sample suspected cases. The case definition adopted by the MoH included suspected and confirmed cases. Patients were suspected of having CHIK if they presented with at least one of the following symptoms or signs: fever (defined as a temperature ≥38°5, measured by a HCW), arthralgias, myalgias, headaches, rash, fatigue, nausea, vomiting, diarrhea, bleeding, or jaundice. A ‘confirmed’ case met the clinical case definition and was PCR-positive.

Patients with suspected CHIK who were identified by the CIRMF team were examined physically, then data were collected on a standardized questionnaire, including age, sex, residence, time of onset and intensity of symptoms, and location of arthralgias (Figure S2). Analgesics and non steroidal antiinflammatory drugs (paracetamol, ibuprofen) were provided to patients according to MoH recommendations. If necessary, patients were hospitalized, and the length of stay was transmitted to our team. Finally, clinical data on the disease course and outcome were collected during a 3-month period.

### Blood collection and laboratory analyses

Blood samples were collected in two 7-ml EDTA Vacutainer tubes and one 7-ml dry tube (VWR International, France). The tubes were stored in the dark at +4°C until arrival at the laboratory.

Thick and thin blood films were stained with 20% Giemsa and examined for malaria parasites. Patients with positive test were excluded from the study. Hematological (Hematology Analyser ACT 10, Beckman Coulter) and biochemical (creatinine, AST, ALT) tests were performed (Automatic Analyser Hitachi model 902, Roche Diagnostics).

For molecular studies, RNA was extracted from 140 µL of plasma by using the QIAamp Viral RNA Mini Kit according to the manufacturer's recommended procedures (Qiagen, Courtaboeuf, France). cDNA was synthesized in a 9700 thermocycler (Applied Biosystems, Foster City, CA, USA), where 25 µL of extracted RNA was mixed with 25 µL of High Capacity cDNA kit (Applied Biosystems, Foster City, CA, USA). Finally, 5 µL of newly synthesized cDNA was used as template in 25 µL of TaqMan Universal PCR Master Mix and then, thermocycled in a 7500 Real-Time PCR system (Applied Biosystems).

RNA positive and negative controls were added in each run. The TaqMan PCR products were identified by curves using a 7500 system SDS software.

The quantitative PCR mix were run with 400 nM of each primer and 200 nM of probe. The E1 gene (208 bp) was targeted for the CHIKV detection (genome position, 10387–10595): CHIK-S (S = Sense): AAGCTYCGCGTCCTTTACCAAG; CHIK-R (R = Reverse): CCAAATTGTCCYGGTCTTCCT; CHIK-P (P = Probe): CCAATGTCYTCMGCCTGGACACCTTT [34]. For DENV detection, the 3′ UTR (107 bp) was targeted (genome position, 10590–10697): DENt-S (t = total, for the 4 serotypes): AGGACYAGAGGTTAGAGGAGA; DENt-R: CGYTCTGTGCCTGGAWTGAT; DENt-P: ACAGCATATTGACGCTGGGARAGACC [35]. The probes used for CHIKV, then for DENV assays were labeled with FAM-reporter and TAMRA-quencher (Applied Biosystems). Quantified RNA transcripts and cell-culture supernatants of CHIKV and DENV were used in 10-fold dilutions as standards for viral load (VL) determination. The VL was determined by comparison to a standard curve. This standard curve was obtained from standard RNA which was diluted at 10 in 10 times. Exponential regression was used to determine the CHIKV viral loads from the threshold cycle. The standard linearity minimum was <10^1^ cDNA genome equivalents/mL. DENVs were typed as previously described [35].

Co-infected patients were excluded from this study.

### Statistical analyses

Statistical analyses were performed using Epi Info software (6.04, Epiconcept). Results were expressed as averages (with their SD) and percentages (with their 95% confidence interval, CI). Student's t test was used to compare laboratory parameters (samples from 50 healthy volunteers recruited at the CIRMF medical unit, with sociodemographic characteristics similar to those of the study subjects, were selected as controls) and continuous clinical variables. For qualitative variables, the Chi square test or Fisher's exact test was used as appropriate. A p value <0.05 was considered to denote statistical significance.

## Results

### Outbreak description

From May to July 2010, 2731 suspected cases were recorded in the two provinces, and 1208 cases (44.2%) were laboratory confirmed (Table 1). There were 1139 CHIKV+ cases (94.2%) in Haut Ogooue province. In all, 933/2063 (45.2%) cases were confirmed in Franceville, representing 81.9% of the confirmed cases in the province.

**Table 1:** Distribution of suspected and confirmed cases during the 2010 Chikungunya outbreak in south-east Gabon.

		Suspected Cases	CHIK+	%
**Haut-Ogooue**	Franceville	2063	933	45.2
	Moanda	169	102	60.3
	Mounana	112	45	40,1
	Bongoville	36	9	25
	Okondja	30	12	40
	Ngouoni	14	10	71.4
	Other	79	28	35.4
**Ogooue Lolo**	Koulamoutou	195	56	28.7
	Lastourville	9	1	11
	Ndangui	24	12	50
**Total**		2731	1208	44.2

**Figure 2:**
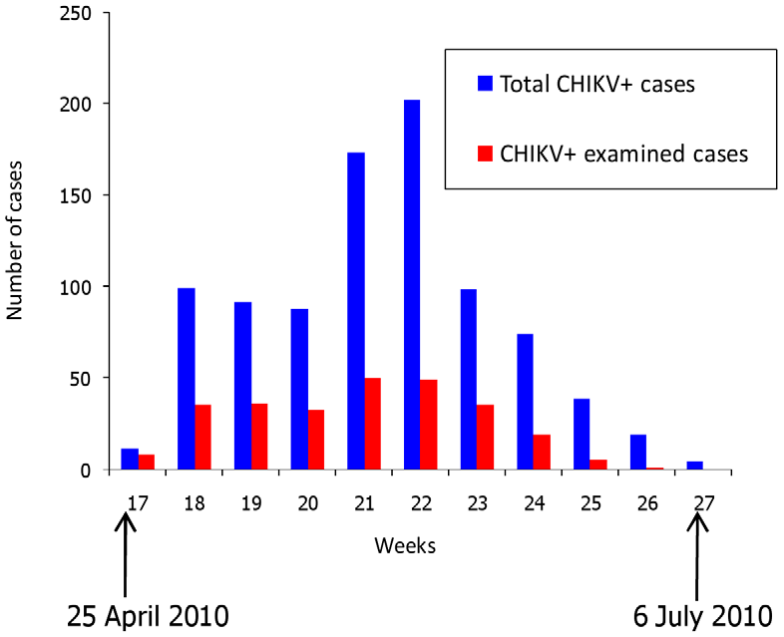
Distribution of Chikungunya confirmed cases according to the date of onset, in Franceville, 2010.

The first cases were confirmed on April 25 (week 17), the peak incidence was reached at weeks 21 and 22 (400 confirmed cases), and the outbreak ended at week 27 (Figure 2).

### Study population

**Figure 3:**
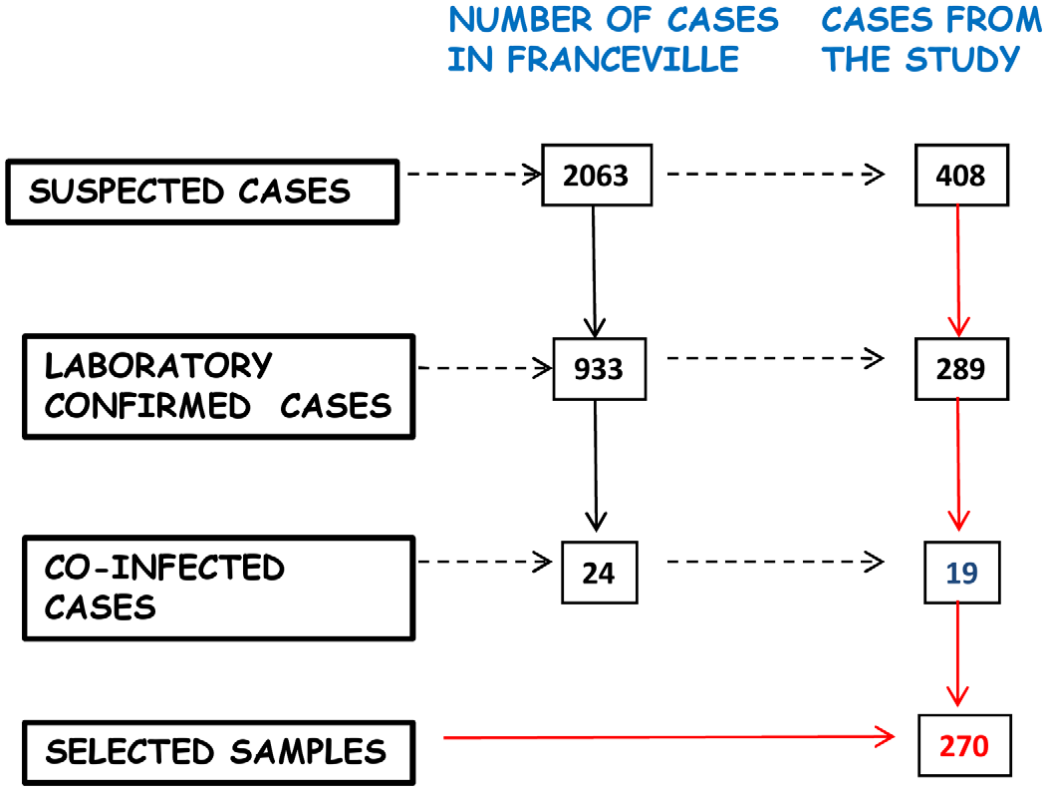
Algorithm for cases selection.

Of the 2063 suspected cases detected in Franceville, 408 (19.8%) were examined by the CIRMF medical team, of whom 289 were CHIKV+, 19 were co-infected (with 18 CHIKV+/DENV+ patients and 1 CHIKV+/DENV+/malaria+). So, 270 CHIKV+ (66.2%) were selected for the study (Figure 3). The 119 CHIKV- patients did not receive a diagnosis.

They represented 28.9% of all CHIKV+ patients in Franceville. They were distributed throughout the outbreak, with 99 (36.6%) patients included at the epidemic peak, in weeks 21 and 22 (Figure 2).

The M/F sex ratio of the study population was 0.85 and mean age was 30±16 years (range, 1–77). Fifty-six patients (20.7%) were under 16 years old, 70 (26.3%) 16–30 years old, 92 (34.1%) 31–45 years old, and 53 (18.9%) over 45 years old.

### Clinical symptoms

CHIK patients consulted an average of 2 days (range, 0 to 18 days) after the onset of symptoms, 232 (85.9%) from day 0 to 3, 30 (11.1%) from day 4 to 7, and 8 (3%) after day 7. The mean duration of symptoms in the acute phase was 7 days (range, 1–24 days). Hospitalization was necessary for 42 patients (15.5%) with more pronounced manifestations, and the mean length of stay was 2.6 days (range, 1 to 6 days).

**Figure 4:**
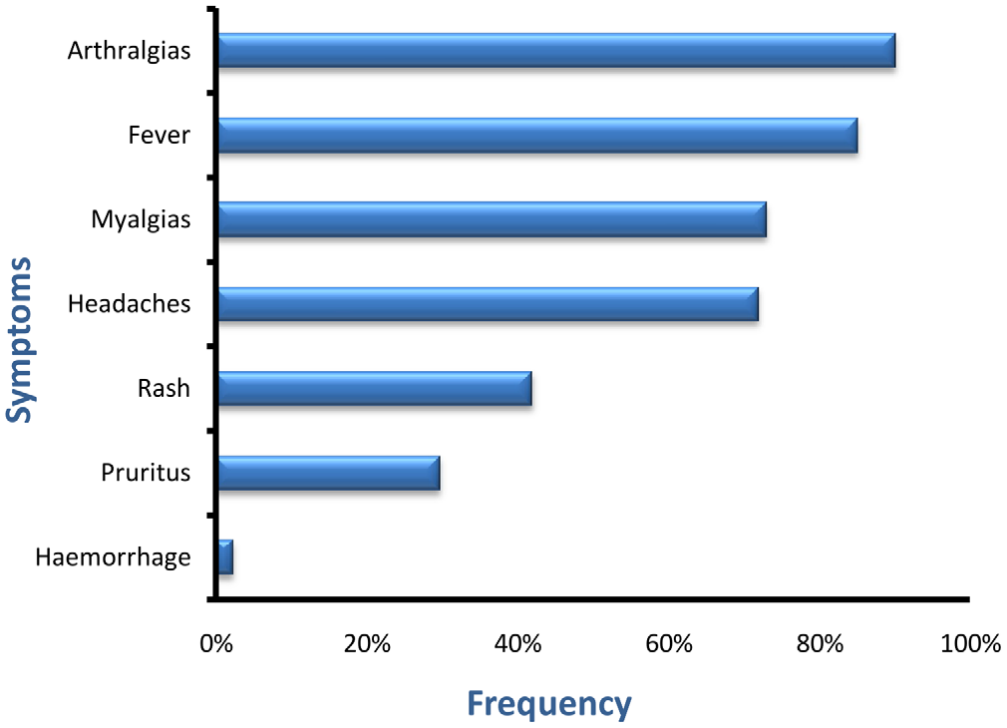
Frequency of the main symptoms in patients with Chikungunya during the 2010 Franceville outbreak.

At the time of the initial consultation, 230 (85%) patients complained of fever, 246 (90.4%) had arthralgias, 197 (72.9%) myalgias and 194 (71.8%) headaches (Figure 4). They all described an abrupt onset of the illness.

**Figure 5:**
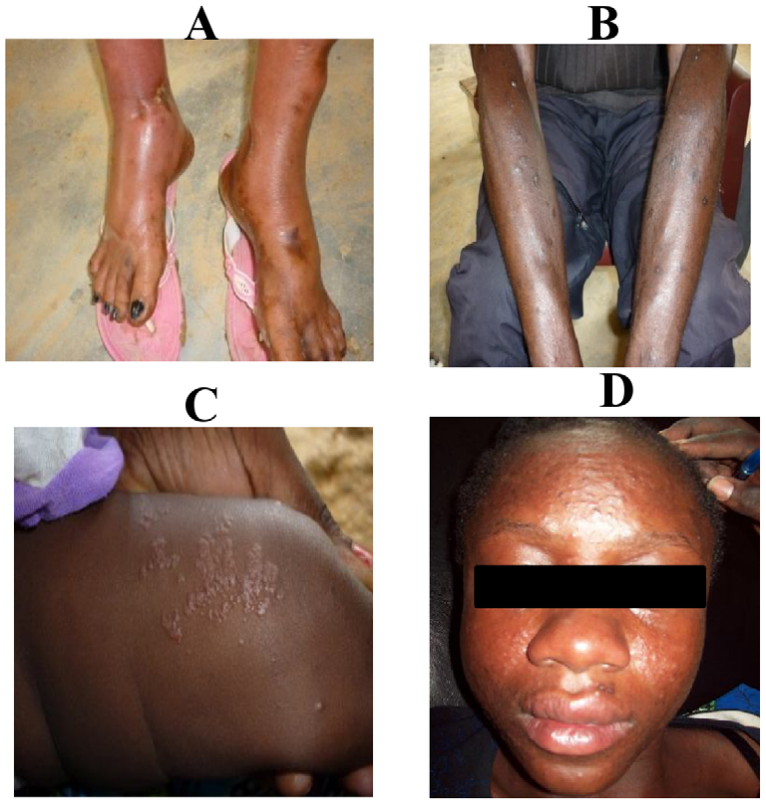
Photographs of clinical manifestations in CHIKV+ patients during the 2010 outbreak in Franceville, Gabon. **A:** Swelling of the ankles and feet in a 43-year-old woman. **B:** Resolutive maculopapular rash of the arms in a 41-year-old man. **C:** Bullous vesicles on the thigh of a 3-year-old child. **D:** Rash with edema of the face in a 16-year-old girl.

Joint pain was mostly polyarticular, bilateral and symmetrical. On average, 7 joints per patient were affected. Among the 246 patients with arthralgias, 100 (40.7%) had fewer than 5 affected joints, and 146 (59.3%) had 5 or more affected joints. Arthralgias occurred in the large joints (shoulders, elbows, wrists, knees, ankles) in 242 (98.4%) patients, and in the lower limbs in 220 (89.4%) patients; the spine was affected in 146 (59.3%) patients. Incapacitation was noted in 158 (64.2%) patients (Table 2), and swelling of the elbows, wrists, knees or ankles was noted in one-quarter of these patients (Figure 5A).

**Table 2:** Location of arthralgias in CHIKV+ patients during the 2010 outbreak in Franceville, Gabon.

		Totaln = 246		Low Viral Loadn = 58		High Viral Loadn = 65		p value
		n	%	n	%	n	%	
**Articular groups**	Large joints	242	98.4	52	89.6	53	81.5	0.6
	Small joints	94	38.2	14	24.1	19	29.2	
**Upper limbs**	Shoulders	71	29	15	25.8	9	13.8	
	Elbows	120	48.8	29	50	21	32.3	0.3
	Wrists	141	57.3	26	44	31	47.7	
	Hands	81	32.9	12	20.7	15	23	
**Lower limbs**	Knees	170	69.1	36	62	35	53.8	
	Ankles	176	71.5	32	55.1	41	63	0.18
	Feet	34	13.8	3	5.2	9	13.8	
**Spine**		146	59.3	31	53.4	31	47.7	
**Intensity**	Severe	158	64.2	27	46.5	15	23	
	Moderate	81	33	22	37.9	18	27.7	0.02
	Minimal	7	2.8	3	5.1	11	16.9	

Low Viral Load: <100 000 DNA cDNA genome equivalents/ml.

High Viral Load: ≥100 000 DNA cDNA genome equivalents/ml.

Myalgias mainly affected the forearms, arms, thighs and calves, and sometimes became increasingly incapacitating. No cases of myositis were seen. Headaches were beating or weighty, and were located in the frontal, parietal, retro-orbital or, rarely, occipital regions. Skin lesions were noted in 113 (41.8%) patients, in the form of macular or maculopapular exanthema (Figure 5B), morbiliform or bullous rash in a few children (Figure 5C), and was accompanied by pruritus in one-quarter of cases. A more aggressive form, with facial edema, was seen (Figure 5D). Peeling of the affected skin occurred a few days after. Digestive symptoms, consisting of abdominal pain, nausea, vomiting and diarrhea (87, 32%), were described, and mild bleeding of the nose and gums reported (6, 2.2%) in patients with normal platelet counts. Seizures occurred in 2 children (CSF was not sampled), who recovered without sequelae.

No complications or deaths were reported.

### Distribution of clinical forms

The disease was classified in four forms according to the existence of fever and/or arthralgias (plus any additional symptoms), namely mixed (fever and arthralgias both present), pure febrile (fever without arthralgias), pure arthralgic (arthralgias without fever), and unusual (neither fever nor arthralgia).

**Figure 6:**
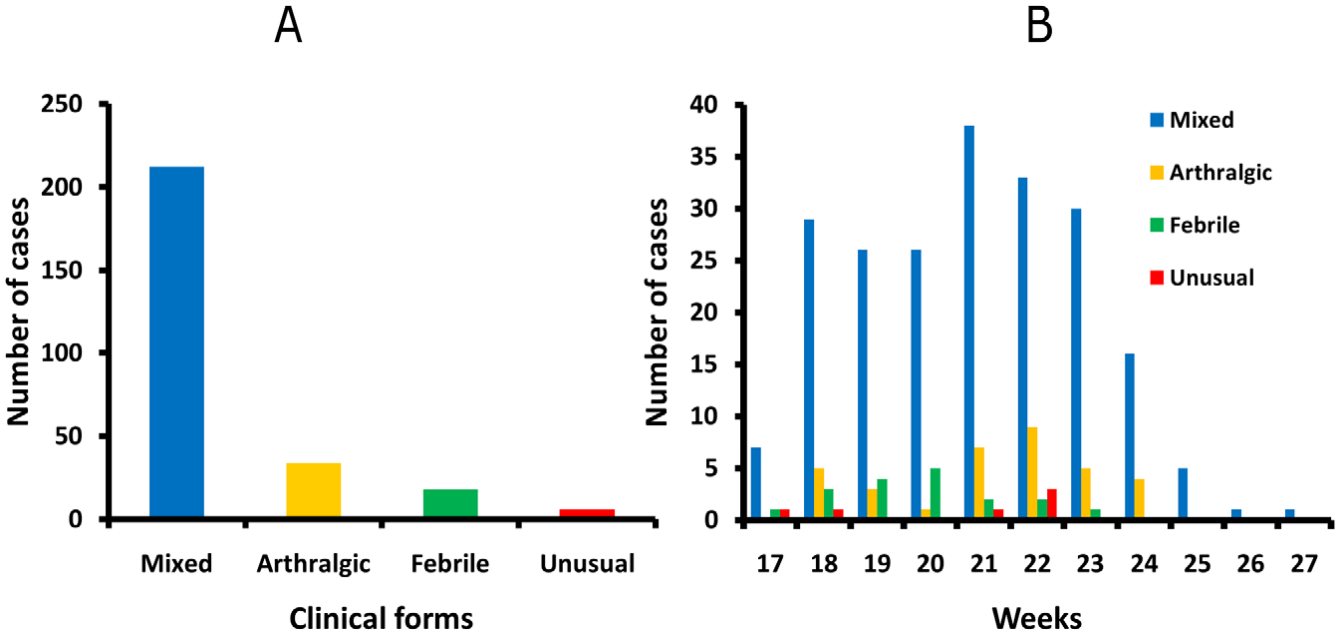
Distribution of clinical forms. **A:** Distribution of the four clinical forms. **B:** Distribution of clinical forms according to the period of the outbreak.

The mixed form was found in 212 (78.5%) patients, the arthralgic form in 34 (12.6%), the febrile form in 18 (6.7%), and the unusual form in 6 (2.2%) (Figure 6A). Patients with the unusual form mainly had digestive symptoms (Table 3).

**Table 3:** Symptoms of patients with unusual forms.

Patients	Age	Sex	Symptoms	Biological disorders	Viral load
**1**	42	Female	Asthenia, myalgias, headaches, nausea	Leukopenia	ND
**2**	29	Female	Asthenia	None	10^1^
**3**	45	Female	Asthenia, nausea, vomiting, diarrhea	None	1.92×10^7^
**4**	1	Male	Vomiting, diarrhea	None	6.63×10^7^
**5**	17	Female	Rash	None	ND
**6**	37	Female	Asthenia, headaches, nausea	None	2.46×10^5^

ND: Not done; Viral load of 1 corresponds to a positive PCR signal detected outside the standard linearity. Viral loads are expressed in cDNA genome equivalents/ml.

The performance indicators of the “fever and arthralgia” combination were estimated in suspected patients (including PCR negative patients), taking into account the true positive (TP), true negative (TN), false positive (FP) and false negative (FN) results. Sensitivity was calculated as TP/(TP+FN), specificity as TN/(TN+FP), positive predictive value (PPV) as TP/(TP+FP) and negative predictive value (NPV) as TN/(FN+TN). The “fever and arthralgia” combination showed a sensitivity and specificity of respectively 73.1% and 41% for PCR positivity, and PPV and NPV of 78.5% and 34.4% (Table 4).

**Table 4:** Performance indicators for the pair “fever and arthralgia” in suspected patients.

	Symptoms evaluated		
	Present	Absent	Total
**Positive test**	212	58	270
**Negative test**	78	41	119
**Total**	290	99	389

Sensitivity = 73.1%; Specificity = 41.4%; Positive Predictive Value = 78.5%; Negative Predictive Value = 34.4%.

All four forms occurred throughout the outbreak, with a majority of patients in the mixed form between week 18 to 23 (Figure 6B).

There was no significant difference in the mean duration of symptoms (p = 0.33) across the 4 clinical forms. Hospitalization was necessary for 35 (16.5%) patients in the mixed form, 3 (8.8%) patients in the arthralgic form, 4 (2.2%) patients in the febrile form and none of the unusual form (p = 0.33), and there was no significant difference in the mean length of hospital stay (p = 0.16).

### Biological parameters

Hematological and biochemical parameters were available for 224 patients. Mean counts of leukocytes (5243±1676/mm^3^, range 1900–11000/mm^3^) and platelets (233 089±81 750/mm^3^, range 52000–455000/mm^3^) did not differ from the controls. Nevertheless, anemia (mean hemoglobin 12.3±1.7 g/dl, range 8–17 g/dl), and lymphocytopenia (mean lymphocyte count 2228±216/mm^3^, range 184–7150/mm^3^) were significantly frequent in CHIKV+ patients (respective p values 0.0009 and <0.0001) than in the controls. Liver enzymes (AST and ALT) and creatinine levels were significantly higher (respective p values 0.03, 0.003 and <0.0001) in CHIKV+ patients than in controls (Table 5).

**Table 5:** Comparison of laboratory findings in patients with acute Chikungunya and healthy controls.

Biological parameters	CHIKV+, n = 224		Controls, n = 50		p value
	Mean	SD	Mean	SD	
Hemoglobin	12.3	1.7	13.2	1.8	0.0009
WBC count, cell/mm^3^	5243	1676	5242	1727	0.99
Lymphocytes count, cell/mm^3^	2228	216	2039	205	<0.0001
Platelet count, cell/mm^3^	233089	81750	219000	66000	0.25
Aspartate aminotransferase level, UI/L	45	35	34	16	0.03
Alanine aminotransferase level, UI/L	33	17	25	19	0.003
Creatinine level, µmol/L	96	32	71	15	<0.0001

There was no significant difference in hemoglobin rate, leukocyte, lymphocyte, or platelet count, or biochemical parameters across the four clinical forms.

### Viral load

A total of 123 patients were selected for viral load (VL) assay. The selection criteria included age, sex, site of consultation, area of residence, week of disease onset, and clinical form. Their characteristics were comparable to those of the initial sample.

The mean VL was 1.2×10^7^ (range, 1–4.4×10^8^). Fifty eight patients (47.2%) had low VL (<100 000 DNA copies per mL, Group 1), and 65 (52.8%) had high VL (≥100 000 DNA copies, Group 2). There was no difference in hemoglobin rate, leukocyte or platelet count or biochemical data between the two groups. However, lymphocytopenia was significantly more frequent (p = 0.01) in group 2 than in group 1. Moreover, there was no difference in symptoms, affected joints (large vs small), their location or intensity (Table 2).

There was no difference in VL according to the day of sampling or symptom onset (day 0 to 3, day 4 to 7, and after day 7). VL did not differ across the four clinical forms.

### Outcome at month 3

Of the 270 CHIKV+ patients included in the study, 225 (83.3%) had completely recovered by day 30. The other 45 patients complained of persistence or relapse of fever (n = 6), arthralgias (n = 36; incapacitating arthragias were still present in 5 patients), myalgias (n = 11), headaches (n = 20), pruritus (n = 5) or fatigue (n = 10).

At day 90, 11 patients had persistent arthralgias. Among the 5 patients with incapacitating arthralgias, 4 recovered by day 90 and one was lost to follow-up. Three patients had headaches.

## Discussion

During a recent concomitant CHIKV/DENV outbreak which occurred in south-east Gabon, we conducted a prospective study in the most affected town, obtaining clinical and biological descriptions of 270 laboratory-confirmed cases of CHIK. We found variable clinical manifestations, including unusual forms.

This is the second large epidemic to be reported in Gabon, after the concurrent CHIKV/DENV-2 intrusion in 2007, in which 2 provinces and 7 towns were affected. International shipping was suspected of providing the portal of entry for both viruses [20]. Inside the country, the infection spread insidiously, along a north-west/south-east axis via the railway and roads, leading to a new outbreak in 2 provinces and 10 towns, 600 km distant and 3 years later. Identified in Gabon just before 2007, in an area where *Aedes aegypti* was previously predominant [36], *Aedes albopictus* was the main vector of the first outbreak [20], and the second. Given the rapid spread of this mosquito, even in temperate areas [18], gradual invasion of the entire country by these viruses is foreseeable.

This descriptive and prospective African study provides exhaustive clinical and biological data than previous outbreaks in the DRC, Republic of Congo, Cameroon and Gabon where descriptions were succinct [7], [19], [20], [37]. Available descriptions have also been made in patients from la Reunion Island [25], [26], notably, in a prospective study [38].

A large number of patients were enrolled in this study. They represented about one-third of all laboratory-confirmed cases, and were distributed throughout the outbreak period, following the global epidemic curve. This series is therefore largely representative of the epidemic, and is as large as two other studies which included respectively 157 and 274 patients [25], [38]. Other studies included less than 100 patients [28], [39], [40]. However, some patients with mild symptoms did not consult at the hospitals, and constituted the unique bias of recruitment. So, the number of enrolled patients is underestimated.

This study did not provided any information on asymptomatic because patients' recruitment occurred in the hospitals (individual with symptoms only). In a recent study, asymptomatic CHIKV infection was estimated at 28% [41]. With regard of the number of CHIKV negative cases, the circulation of other arboviruses during this epidemic cannot be ruled out. During the 2007 outbreak, DENV-2 was detected [20] and a fatal case of West Nile virus infection was diagnosed in Libreville [42]. Moreover, recent sero-epidemiological studies have suggested the circulation of DENV, West Nile and Rift Valley Fever viruses in rural populations [43]–[45].

We observed marked clinical variability. As previously described, fever and polyarthralgias were the most frequent manifestations, affecting nearly all the patients [3], [14], [22], [25], [38], [41]. They were found in respectively 85% and 90% of our patients, and were both present in three-quarters of cases. We found a weak association between the “fever and arthralgia” combination and PCR positivity, with a sensitivity and specificity of 73.1% and 41%, while in another recent study this combination had a diagnostic sensitivity of 84% and a specificity of 89% in an epidemic setting [41]. Differences in the case definition and the assay could explain this discrepancy.

In its classical form, CHIK is a painful febrile illness characterized by incapacitating arthralgias. Patients presenting with a bent gait due joint pain, mainly affecting the wrists and ankles, are easily recognizable. Incapacitating arthralgias are considered pathognomonic for the disease. When the symptoms are less severe, the clinical diagnosis becomes less clear-cut, and they may also be seen in other diseases such as malaria, DF and typhoid fever. To date, only the classical DF has been reported in Africa. To our knowledge, there is no comparative study of the two diseases. Complications such as dengue hemorrhagic fever and dengue with shock syndrome and persistent arthralgia following CHIK constitute the noteworthy differences. In our field experience, these diseases are clinically indistinguishable, and the problem of differential diagnosis may be compounded during concomitant CHIKV/DENV outbreaks in a malaria endemic area.

Pure febrile and arthalgic forms were also seen, adding to the clinical variability. The other symptoms were as frequent as in previous studies [14], [25], [38], [41]. Myalgias sometimes resulted in incapacity. Rashes were easily diagnosed in our darker-skinned population, and uncomplicated bullous lesions were also seen in children. The mechanism by which bleeding occurs despite normal platelet counts is unclear, but this classifies CHIK in the viral hemorrhagic fever group, in this area where ebolavirus and yellow fever virus also circulate.

We identified 2.2% of patients who had neither fever nor arthralgias. These unusual forms were evoked in the Reunion Island. This is their first description due to a strong clinical presumption of physicians and a less sensitive and specific case definition. Furthermore, no severe forms (requiring maintenance of vital function) and deaths as described in the Reunion Island [14], [26] were noted in Gabon. In a context of frequent self-medication, as in our study, many patients with unusual and non severe forms did not consult a health service, and some of those who did may have been misdiagnosed. So, the total number of cases, in this epidemic, and the proportion of unusual forms, are probably underestimated. Our findings imply that, during epidemic periods, clinicians should not focus solely on fever and arthralgias.

The four clinical forms were present throughout the outbreak period, suggesting that pathogenicity did not vary markedly.

We also observed rare relapses or persistence of arthralgias, as previously described [3], [14], [22]. The low rate of persisting arthralgia in our sampling contrasts with previous studies [46]–[48]. The little number of individuals followed up and the low mean age (at 30 years) of our sampling could explain it. In previous studies, the mean age of the population study was higher (at 50 years) and the incidence of persistent arthralgia was higher in older patients [46]–[48]. Immunonologic and genetic factors (anti CCP antibodies, antinuclear antibodies and HLA DR alleles) are associated with rheumatoid arthritis following Chikungunya fever [49]. Their absence in our patients could also explain low rate of persistent arthralgia.

In CHIKV infections, biological abnormalities are varied, transient and nonspecific, as in many other viral diseases [25], [38]. In our study, biological data from CHIKV+ patients were compared with those of healthy volunteers than CHIKV-, in order to avoid a bias in results interpretation. CHIKV- patients were considered as having another disease. Varied biological abnormalities were found. Anemia may have been due to parasitic coinfection (ankylostomiasis, ascariasis). Lymphocytopenia correlated with viremia, as recently described [38], and is probably due to excessive apoptosis with peripheral lymphocyte destruction. The increase in liver enzyme and creatinine levels may have been due to drug toxicity (particularly when ibuprofen and paracetamol are associated), muscle damage or even rhabdomyolysis. Finally, the disease severity did not correlate with VL in our study, contrary to a recent study in which the severity criteria were different [50]. Together, these findings imply that symptoms are not directly related to the virus but rather to the immune reaction.

Theoretically, PCR is the preferred method for detecting and quantifying CHIKV viral RNA, mainly during the first week after infection [4], [5]. The detection of the virus later than 7 days after symptom onset in 3% of our patients is surprising. It is conceivable that their initial symptoms were due to another disease such as malaria or dengue, before they contracted CHIKV.

The genotype of CHIV implicated in Gabonese and Cameroonian outbreaks belongs to the ESCA phylogroup and harbor the A226V mutation [20]. This mutation has also been found in samples from La Reunion Island which the genotype belongs to the Asian phylogroup. This mutation improves replication and transmission efficiency in *Aedes albopictus* mosquitoes [6]. To date, no genotype and no mutation have been found associated with specific clinical forms.

Finally, our study showed that this outbreak, as the first one, occurred during the rainy season and *Aedes albopictus* was the main vector, with an extension in other parts of the country. Globally, the clinical picture did not differ from Asian, European and Indian Ocean Islands studies. The acute phase and outcome seemed similar, as biological abnormalities and treatment efficacy. In the other hand, there were some noteworthy distinctive features: the mean duration of symptoms in the acute phase is long (7 days reaching to 24 days), relapses and persistent arthralgia were rare, there were no severe forms or deaths, and unusual forms were well described.

Epidemiological surveillance must continue in order to detect any change in the pathogenicity of CHIKV.

## Supporting Information

Figure S1 Research authorization.
(PDF)

Figure S2 Standardized questionnaire.
(PDF)

Checklist S1 Strobe checklist.
(DOC)
