# Supplementary material for: Clinical Forms of Chikungunya in Gabon, 2010
Source: PLoS Negl Trop Dis. 2012 Feb 14;6(2):e1517. doi: 10.1371/journal.pntd.0001517 (PMC3279511; doi:10.1371/journal.pntd.0001517)
Supplement: Figure S1 — Research authorization. (PDF) [file pntd.0001517.s001.pdf]

MINISTERE DE LA SANTE, DES AFFAIRES SOCIALES, DE LA SOLIDARITE ET DE LA FAMILLE.

SECRETARIAT GENERAL

DIRECTION REGIONALE DE SANTE SUD-EST

✉ : 528 ☎/🖷 : 67-72-62

FRANCEVILLE

REPUBLIQUE GABONAISE

UNION-TRAVAIL-JUSTICE.

N° ....189............./DRSSE

AUTORISATION

Dans le cadre de la gestion de l'épidémie de Chikungunya qui sévit actuellement dans la Province du Haut Ogooué, je soussigné, Jean baptiste ATSOUGOU, **Directeur Régional de la Santé Sud Est**, autorise l'appui technique et logistique du CIRMF par le biais de l'Unité des Maladies Virales Emergentes.

Cet appui sera basé sur la surveillance épidémiologique, la récupération d'échantillons sanguins pour le diagnostic biologique des cas suspects, et la prise en charge clinique des malades, en collaboration avec les personnels des différents services de santé de la Province.

La présente autorisation est établie pour servir et valoir ce que de droit.

Fait à Franceville, le 27 Mai 2010

Le Directeur Régional de la Santé Sud-Est

DIRECTION REGIONALE DE SANTE SUD-EST FRANCEVILLE ★ Le Directeur ★ UNION-TRAVAIL-JUSTICE

Jean Baptiste ATSOUGOU
