# Supplementary material for: Clinical Forms of Chikungunya in Gabon, 2010
Source: PLoS Negl Trop Dis. 2012 Feb 14;6(2):e1517. doi: 10.1371/journal.pntd.0001517 (PMC3279511; doi:10.1371/journal.pntd.0001517)
Supplement: Figure S2 — Standardized questionnaire. (PDF) [file pntd.0001517.s002.pdf]

IDENTIFICATION DES INFECTIONS ARBOVIRALES
AU GABON

FICHE DE RECUEIL DES DONNEES

Etablissement sanitaire : …………………………….

Date d'inclusion…………./__/__/ /__/__/ /__/ __/

IDENTITÉ

Nom…………………………………………..Prénom……………………………………………………….
Age……………………………………………… [__]
Sexe…………………☐M ☐F [__]
Profession…………………………………………………………………………………………..[__]
Domicile ……. …………………………………Téléphone……… ………….………….............…………....[__]

ANAMNÈSE

Date de début des symptômes……………………………………………………………………[__]

	Absente	Minime	Modérée	Importante (Intensité)		
Fièvre…………………	☐	☐	☐	☐	Température………..°C	[__]
Asthénie………………	☐	☐	☐	☐		[__]
Myalgies……................	☐	☐	☐	☐		[__]
Céphalées…………….	☐	☐	☐	☐		[__]
Arthralgies:…………..	☐	☐	☐	☐		[__]

Entourer le siège des Arthralgies [__]

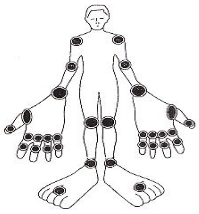

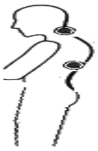

,

Digestifs

	Absente	Minime	Modérée	Importante (Intensité)		
Nausées…………..	☐	☐	☐	☐		[__]
Vomissement……...	☐	☐	☐	☐		[__]
Diarrhées………….	☐	☐	☐	☐		[__]
Eruption cutanée……...	☐	☐	☐	☐	Préciser le type si présente	[__]
Hémorragie………..	☐	☐	☐	☐	Préciser le type si présente	[__]

Autres signes fonctionnels (à préciser)……………………………………………………………

ÉXAMEN CLINIQUE

Signes généraux :

Etat général……………………☐ Bon ☐ Mauvais
Conjonctives……………………………..
T.A…………………..mmHg
Pouls………………….bpm

Examen neurologique

Score de Glasgow**………./15** [__]
Troubles moteurs………………………☐ Oui ☐ Non Si oui, préciser
Troubles des réflexes(ROT/Babinsky)...☐ Oui ☐ Non
Raideur méningée/Photophobie………..☐ Oui ☐ Non [__]
Raideur de la nuque……………………☐ Oui ☐ Non [__]

Autres:……………………………………………………………………………………………..
:……………………………………………………………………………………………..
……………………………………………………………………………………………..
……………………………………………………………………………………………..
:……………………………………………………………………………………………..
:……………………………………………………………………………………………..
……………………………………………………………………………………………..

DONNEES DU LABORATOIRE

NFS : GB…………....../mm³ Neutro(%)…………..Lympho(%)……………………………………………………………..[__]
GR:……………/mm³…….Hb……………g/dl… Plaquettes…………/mm³…VS……………………………………
GE :……………………………………………………………………………………………..........……[__]
GOT…………..GPT…………………………………….
Urée……………..........Créatininémie…………………..Acide urique……………………………………………………….

LCR……………Glyco/Protéinorachie………………...Cytologie…………N(%)……………L(%)…………………...……[__]

TRAITEMENT

…………………………………………………………………………………………..............................
………..
………..
………..

DIAGNOSTIC VIROLOGIQUE

……….[__]
……….
………..

OBSERVATIONS……………………………………………………………………………………
………..
